# Prospects for the Analysis and Reduction of Damaging Behaviour in Group-Housed Livestock, With Application to Pig Breeding

**DOI:** 10.3389/fgene.2020.611073

**Published:** 2020-12-23

**Authors:** Laurianne Canario, Piter Bijma, Ingrid David, Irene Camerlink, Alexandre Martin, Wendy Mercedes Rauw, Loïc Flatres-Grall, Lisette van der Zande, Simon P. Turner, Catherine Larzul, Lotta Rydhmer

**Affiliations:** ^1^GenPhySE, INRAE French National Institute for Agriculture, Food, and Environment, ENVT, Université de Toulouse, Toulouse, France; ^2^Animal Breeding and Genomics, Wageningen University & Research, Wageningen, Netherlands; ^3^Institute of Genetics and Animal Biotechnology, Polish Academy of Sciences, Warsaw, Poland; ^4^Department of Animal Breeding, National Institute for Agricultural and Food Research and Technology, Madrid, Spain; ^5^AXIOM, La Garenne-Colombes, France; ^6^Adaptation Physiology, Wageningen University & Research, Wageningen, Netherlands; ^7^Topigs Norsvin Research Center B.V., Beuningen, Netherlands; ^8^Scotland's Rural College, Kings Buildings, Edinburgh, United Kingdom; ^9^Department of Animal Breeding and Genetics, Swedish University of Agricultural Sciences, Uppsala, Sweden

**Keywords:** aggression, genetics, savaging, social interactions, *Sus scrofa*, model, breeding, tail biting

## Abstract

Innovations in the breeding and management of pigs are needed to improve the performance and welfare of animals raised in social groups, and in particular to minimise biting and damage to group mates. Depending on the context, social interactions between pigs can be frequent or infrequent, aggressive, or non-aggressive. Injuries or emotional distress may follow. The behaviours leading to damage to conspecifics include progeny savaging, tail, ear or vulva biting, and excessive aggression. In combination with changes in husbandry practices designed to improve living conditions, refined methods of genetic selection may be a solution reducing these behaviours. Knowledge gaps relating to lack of data and limits in statistical analyses have been identified. The originality of this paper lies in its proposal of several statistical methods for common use in analysing and predicting unwanted behaviours, and for genetic use in the breeding context. We focus on models of interaction reflecting the identity and behaviour of group mates which can be applied directly to damaging traits, social network analysis to define new and more integrative traits, and capture-recapture analysis to replace missing data by estimating the probability of behaviours. We provide the rationale for each method and suggest they should be combined for a more accurate estimation of the variation underlying damaging behaviours.

## Introduction

Pigs are intelligent and explorative animals. However, in modern husbandry they are kept in environments where there is very little more than other pigs to explore. Around 30-70% of European pig farms have problems with tail biting (European Food Safety, 2007). In most countries tail docking is commonly used to reduce biting behaviour despite the EU directive expressly prohibiting this practice (Council directive 2008/120/EC; European Union, 2009). The tail is not the only part of the animal that can attract another pig's interest; ears and other body parts can also be damaged through intentional or non-intentional biting. Ear biting is frequent in certain populations (van Staaveren et al., 2018) and may be more frequent when tails are short-docked (Goossens et al., 2008). These oral, damaging behaviours are mostly associated with young growing and finishing pigs. In gilts and sows, vulva biting occurs, especially in systems where the animals compete for access to a feeder or drinker (Jensen et al., 1995; Rizvi et al., 1998). Vulva biting is connected with frustration at a lack of foraging activity and concerns animals with low rank order in their access to feed. Vulva biting can also be aggressive in nature (Rizvi et al., 2000).

Regardless of the production system, pigs are mixed at some point in life: after birth to equalise litters, at weaning, at the start of the growing phase, as replacement gilts are integrated into the breeding herd and on the way to or at the abattoir. Gilts and sows are also mixed after mating or after the weaning of piglets. Pigs fight when they encounter pigs that are unknown to them (Peden et al., 2018). Aggression is a natural behaviour and establishes dominance relationships (Meese and Ewbank, 1973) but it impairs welfare and reduces productivity if it is performed intensely and/or repeatedly over long periods of time. Thus, common pig production routines lead to compromise of several of the Five Freedoms associated with animal welfare (Brambell, 1965).

Some sows kill their newborn piglets. This savaging behaviour is associated with endocrinal changes in the sows at farrowing (Gilbert, 2001) and the pain of the farrowing process. It is also influenced by the environment, and by the sow's experience. Around 5% to 10% of primiparous sows savage their piglets (Knap and Merks, 1987; van der Steen et al., 1988; Gäde et al., 2008). In older sows savaging is rarer (Gäde et al., 2008). Selection against savaging is probably an ongoing feature of all breeding programmes, with early removal of problematic sows, since even if some piglets survive it, most farmers will avoid gilts born by savaging sows when seeking replacement animals.

Most of the damaging behaviours have a multi-factorial origin: environmental, nutritional and genetic causes all contribute (e.g., Taylor et al., 2012), as illustrated by the bucket model of Bracke et al. (2018). The environment has a major effect on the prevalence of damaging behaviours (Moinard et al., 2003). For example, straw reduces ear and tail biting (Fraser et al., 1991). However, the provision of a healthy and enriched environment does not always prevent such problems. An animal's genotype influences these behaviours as well, and therefore genetic improvement might offer a solution in two ways: by limiting directly (by targeting aggressors/biters) or indirectly (by targeting victims) the mutilations perpetrated by conspecifics.

The environment and genetic status should not be regarded as separate factors. Genotype by environment interaction (GxE) is known to occur when two or more genotypes respond differently to the same environmental change. GxE may influence behavioural traits, and it was found in sows in relation to savaging by Baxter et al. (2011) and in finishing gilts in relation to tail-biting by Canario and Flatres-Grall (2018). Another large-scale study showed that the effects of genotype were additive to environment effects where biting behaviours (oral manipulative behaviours and tail damage) were concerned, showing that genetic improvements and environmental enhancement can complement each other (Camerlink et al., 2015).

Measuring behavioural traits remains challenging despite technological advances in the field of automated recording. Injured animals can be identified by repeated visual monitoring, a method that is highly time-consuming and only provides estimates of infrequent, sporadic or unpredictable behaviours. Lesions on the tail, ears, vulva or skin identify the victim but not the biter, and it is the latter that is of particular interest in breeding against the behaviour. Identifying biters is very complicated, as there is wide variation in their characteristics and a lack of reliable predictors of engagement in biting activity (reviewed by Prunier et al., 2019). In spite of time-consuming recording, some genetic parameters have been estimated not only for receiving, but also for performing damaging behaviour; see Canario et al. (2013) for a review. However, substantial gaps in our understanding of the genetic determinants of these behaviours remain. Further, the damaging behaviours differ in their expression over time. Thus, tail biting can be initiated by one pig and copied by others, and it can spread within and between pens in a manner reminiscent of a disease epidemic (Fraser, 1987; D'Eath et al., 2014; Chou et al., 2019). To understand harmful behaviours it is therefore necessary to move away from the study of these behaviours at a single moment in time, and to undertake longitudinal studies. Increasingly, novel technical developments, such as image-based and sensor-based data analysis methods that automate behavioural recording, will open up opportunities for new selection traits if they are complemented by the automated individual identification of animals (Rodenburg et al., 2019).

## Our Aim

In spite of the considerable research that has been done to date, pigs are still tail-docked in most countries, and an unacceptable proportion of them continue to severely injure their conspecifics in today's production systems (De Briyne et al., 2018). Damaging behaviours, in addition to being unacceptable at both ethical and social levels, raise issues of sanitation and food safety, and they may result in significant economic loss. For example, tail biting in tail-docked finishing pigs costs about €2 per pig in an industry with very low profit margins (D'Eath et al., 2016). The area of this article is quantitative genetics. For recent reviews of molecular genetic studies of damaging behaviour, see Brunberg et al. (2016) and Kasper et al. (2020). We first describe the evidence for genetic effects and genotype × environment interactions influencing damaging behaviour. We then propose and discuss new statistical methods to analyse these behaviours. Finally, we present new strategies that can be implemented in practical breeding. Our analyses refer to the use of genetic models to detect the heritable impact of one individual on other group members, the use of social network analysis to define new and more informative phenotypes for breeding, and the use of capture-recapture methods to overcome barriers currently presented by missing behavioural data. Additionally, we consider the possible synergistic benefits of combining these models.

## Introduction to the Models

The methods used in genetic analyses have been developed from models with one genetic effect (the direct effect of the animal's genotype on its behaviour) to models with two genetic effects, i.e., the direct effect of the animal and a social effect describing the genetic influence of an individual on the phenotype of its group members. The initial social model proposed by Griffing (1967) and Moore et al. (1997) was improved to obtain direct and social breeding value estimations for each individual in the group (Muir, 2005; Bijma et al., 2007). The model with only one genetic effect (the direct effect) is suitable for studying the behaviour of the focal animal alone (e.g., a sow savaging her piglets), whereas the model including both direct and social effects is suitable for studying traits that are affected by social interaction among individuals.

In pigs, studies using the social model have primarily targeted performance traits such as growth rate (e.g., Canario et al., 2017), the assumption being that social interaction among group mates shape individual performance. The social model has also been applied to health traits (Lipschutz-Powell et al., 2012) and injuries caused by damaging behaviour, with the direct effect being connected to the victim and the social effect connected to group mates as potential biters (Canario and Flatres-Grall, 2018). If applied to a behaviour, the direct effect will refer to the individual showing the behaviour and the social effect will refer to the individuals receiving the behaviour.

The social model results in breeding values for a pig's ability to influence the performance or behaviour of group mates, but it does not describe differences in the strength or type of interactions between pairs of pigs within the group. Social network analysis accounts for both direct interactions between animals (e.g., pig A bites pig B) and indirect interactions (e.g., A bites pig B, B bites pig C, and therefore A's behaviour has an impact on C via the intermediate B), as described by Büttner et al. (2015a,b). It quantifies the position of individuals in a social context and the information flow within the network using the degree and strength of interactions (Wasserheit and Aral, 1996; Goh, 2002; Flack et al., 2006). Social networks can be studied and described in a genetic context when pedigree information is combined with outputs from network analysis (Foister et al., 2018).

Damaging behaviours in a group evolve over time. As mentioned above, tail biting shows similarities with the spread of infectious disease (Bracke et al., 2018), as the number of biters increases rapidly after one animal initiates the behaviour. An individual's vulnerability to being tail-bitten is analogous to disease susceptibility, and its propensity to bite another pig is analogous to infectivity. Since we lack the quantitative genetic models to study social interactions and injuries over time, this paper proposes a new model designed for this very purpose, borrowing from models of transmission of infectious disease and from survival time analysis (e.g., Ducrocq, 1994; Anche et al., 2014; Lipschutz-Powell et al., 2014; Biemans et al., 2017). In this model, the amount of tail biting that an individual is subjected to is modelled as a continuous function of time with a Poisson process. The combination of this contagious longitudinal model with the social model should provide better estimates of the direct and social breeding values for tail-biting.

At a higher level of complexity, the roles of animals can change over time. A pig can move from being a non-biter at one stage of production to being a biter at the next (Ursinus et al., 2014), and the probability that an individual will bite or receives bites may depend on the history of biting in the group. To be able to describe this process of change, a method that accounts for missing observations is needed. Missing observations are common in behavioural studies. They are even more numerous when social interactions are considered and involve scattered and rapid movements of animals, as is the case with damaging behaviours. Capture-recapture analysis (CRA) was originally developed to study survival dynamics in wild animal populations (Pradel et al., 1997, 2005). Nowadays CRA has applications in many fields of research, including animal activity patterns (Langrock et al., 2012) and movements (Vogel et al., 2020). The principle of this method of survival analysis in wild species is to use data from several independent but overlapping samples to estimate a probability of survival despite the fact that there is missing information. However, this assumes that a substantial proportion of the animals are captured on several occasions. Applied to behavioural studies, the CRA can be effective in replacing missing records by predicting unobserved values through the calculation of probabilities of a given state. In the present investigation, being a biter of a conspecific or, conversely, a victim are the two studied traits. We propose that the method should be combined with any of the above-mentioned models to identify biters and victims with greater accuracy.

## The Genetic Background of Damaging Behaviours in Pigs

### Tail, Ear, and Vulva Biting

In pigs, tail, ear and vulva biting are prominent forms of abnormal behaviour. Although these behaviours are multifactorial, the primary trigger of redirected behaviour is a barren environment that prevents the normal outlet of strong foraging motivation (e.g., D'Eath et al., 2014). The foraging activity is then directed towards elements in the environment that are most readily manipulated, and this includes the ears and tails of other pigs (van Putten, 1969). Often, these behaviours are not accurately regarded as a form of aggression; they become such when they are used by a pig as a means of displacing others from the feeding area (Prunier et al., 2019).

Breed differences in the tendency to tail and ear bite have been reported (Westin, 2000; Breuer et al., 2003; Sinisalo et al., 2012) but are sometimes not found (Lund and Simonsen, 2000; Guy et al., 2002). The genetic basis of ear and vulva biting is yet to be studied. With use of a direct model, Breuer et al. (2005) estimated a heritability of 0.05 for tail biting behaviour in Landrace pigs expressed on a binary scale, together with a heritability of 0.27 on the underlying continuous scale. The heritability was not statistically different from zero in Large White pigs (Breuer et al., 2005). Only a small number of pigs (3.3%) exhibited tail biting in this study. Canario and Flatres-Grall (2018) observed heritability of 0.06 for tail-biting receipt as measured by the presence of tail injuries in females (on average 7.1% of prevalence in the population) from a composite Sino-European line. Single nucleotide polymorphisms (SNPs) have been identified that are shared by tail biters and victims of tail-biting and differ from the SNPs of pigs in the same pen that are not involved in this behaviour (Wilson et al., 2012). Brain gene expression studies also suggest that biters and victims have more in common than pigs not involved in such activity (Brunberg et al., 2013a,b).

### Aggressive Behaviour Towards Piglets

The savaging of piglets is a highly problematic form of aggression. Up to 15% of primiparous sows savage their piglets (Knap and Merks, 1987; van der Steen et al., 1988; Quilter et al., 2007; Chen et al., 2008). Vangen et al. (2005) observed that Finnish Landrace sows were more aggressive towards their piglets than Finnish Yorkshire sows. Knap and Merks (1987) found that Duroc sows were more aggressive towards piglets than Landrace sows, and that crossbreds were more aggressive than purebred Landrace and Duroc sows. Sow aggression towards piglets is heritable (h^2^ = 0.08–0.90, Knap and Merks, 1987; Grandinson et al., 2003). The heritability for savaging has been reported to vary between 0.20 and 0.90 (Canario et al., 2013). Savaging has also been observed in farmed wild boar sows (Harris et al., 2001). Baxter et al. (2011) found that in a line selected for high piglet survival (as a sow trait) gilts from the high-survival line raised outdoors had a higher frequency of savaging (relative to a control line) when they farrowed indoors.

### Aggressive Behaviour at Other Stages of Production

The high frequency, duration and intensity of aggression at mixing largely results from the sudden grouping of unfamiliar pigs in an environment that is either unsuitable for clear submission or lacks enough space to escape from an attack. Repeated fights outside of mixing periods maintain dominance and control access to food (Fraser, 1984). Pigs show stable individual differences over a period of several months in their propensity to be aggressive (e.g., D'Eath et al., 2010; Horback and Parsons, 2016). The heritability of aggressive behaviour at mixing varies between 0 and 0.44 in weaners, growers, replacement gilts and mature sows (Løvendahl et al., 2005; Turner et al., 2006, 2009; Stukenborg et al., 2012; Appel et al., 2016; Scheffler et al., 2016). The analysis of dyadic encounters between gestating sows in a test arena through a matrix of social interactions yielded higher heritability for being an aggressor (h^2^ = 0.22) than it did for being a victim (h^2^ = 0.05) (Løvendahl et al., 2005). In younger pigs, the act of bullying was more heritable than receipt of it (h^2^ = 0.01–0.08) (Turner et al., 2009), but moderate heritabilities have been reported for receipt of aggression too (e.g., 0.37; Scheffler et al., 2016). Aggression among pregnant sows during washing before farrowing can be frequent (18%) and is moderately heritable (h^2^ = 0.32, Hellbrügge et al., 2008). As a proxy measurement of longer-term aggression, recorded several weeks after regrouping, number of skin lesions has also been found to be heritable (h^2^ = 0.16–0.43; Turner et al., 2009; Desire et al., 2015; Wurtz et al., 2017).

### Genetic Relationships Between Damaging Behaviours

**Figure 1:**
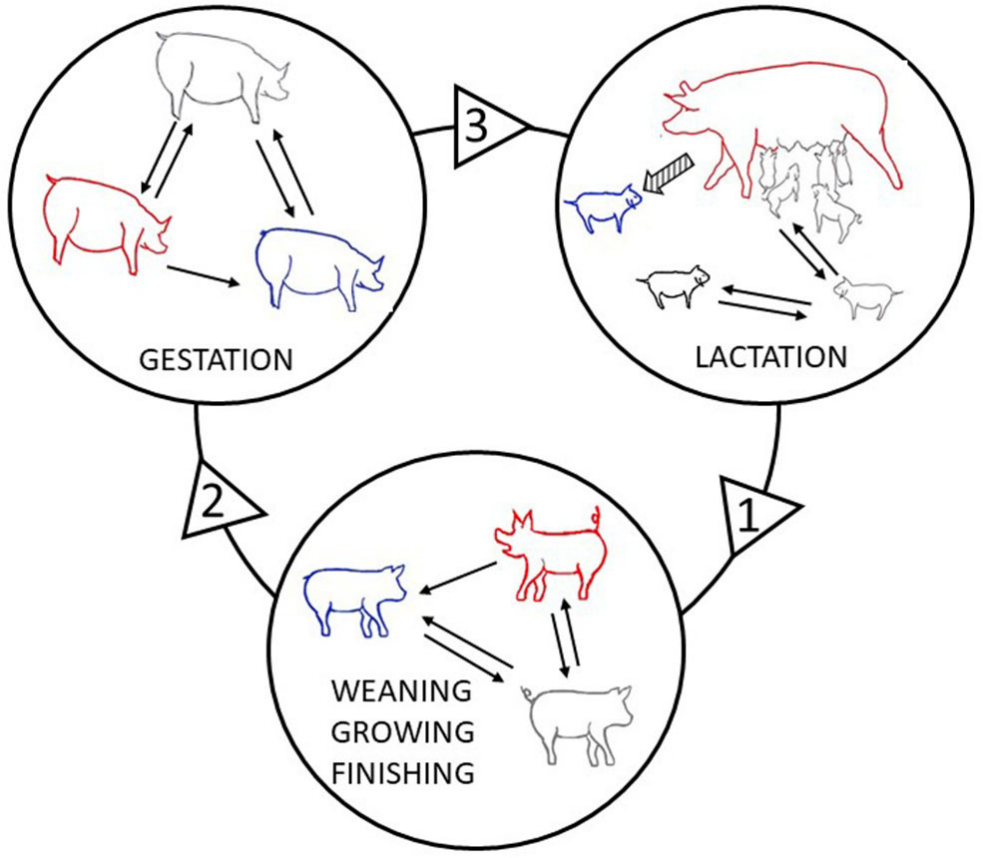
Social interactions in the circle of life: dynamics of social interactions during the life of a female pig. This scheme illustrates how a focal pig (red) interacts with conspecifics at the different stages of its life. Arrows between circles indicate paths from one group to the next as pigs are regrouped when entering a new stage. 1. During the lactation phase, the piglet develops social skills in contact with littermates. When it is moved to the growing and finishing facilities, the piglet is mixed in a pen with both familiar and unfamiliar pigs, which may be relatives or not, and this induces the acute aggression necessary to establish dominance relationships, eventually followed by chronic aggression. Tail and ear biting may also occur at this stage. The focal pig is a biter (red) of a conspecific victim (blue). The pig in grey is neither an aggressor/biter nor a victim. 2. When mature, the female pig, as a gilt, enters a gestation group where it is mixed again with both familiar and unfamiliar gilts, which may be relatives or not. Negative social interactions may occur again, including vulva biting which is typical of this phase. 3. At the end of the gestation phase, pregnant sows are moved to the lactation unit, where they are usually kept in an individual pen or crate. As a dam, the sow develops social interactions with its progeny and may express damaging behaviour towards piglets, including savaging during the first few days after farrowing (hatched arrow). The piglets, influenced by social interactions with their dam and littermates, move on to the next stage of development.

The success of selective breeding strategies to limit damaging behaviours is likely to depend heavily on whether some of these behaviours are governed by the same pool of genes. Genetic studies have until now targeted only one behaviour at a time. Multi-trait genetic analyses can be used to study the relationships between damaging traits. Tail biting and ear biting tend to occur on the same farm, and there is evidence that certain pigs are responsible for performing a disproportionate amount of both behaviours (Brunberg et al., 2011). Both traits are probably stimulated by the same impoverished environmental conditions (Smulders et al., 2008). To our knowledge, the extent to which tail and ear biting are genetically correlated has not been estimated. It is also unknown whether pigs that tail or ear bite when young are later (at a higher age) responsible for tail biting, vulva biting or for the savaging of piglets (Figure 1). There could be a potential link between fighting and savaging if pigs that fight often are also responding to the novelty of unfamiliar group mates. Also, access to feed is believed to be a trigger for some cases of tail biting and is a major reason for vulva biting (Anil et al., 2006). Some degree of association between these two damaging behaviours in the growing-finishing and gestating phase may therefore occur. Selective breeding would benefit from the early detection of such damaging activity.

### Genotype by Environment Interactions Influence Damaging Behaviours

**Figure 2:**
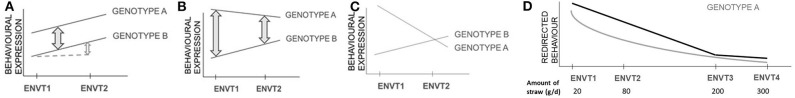
Graphical illustration of genotype by environment interactions (GxE). No genotype by environment interaction (GxE) occurs in the situation with two genotypes with a conserved discrepancy of performance among environments (black lines) **(A)**. Different forms of GxE exist, with quantitative differences in the discrepancy in performance among environments without inversion in ranking **(B)**, and re-ranking of genotypes according to their performance in different environments **(C)**. GxE effects leading to re-ranking are particularly relevant in animal breeding because they modify the relative productive performance of genotypes as measured in two or more environments, making some genotypes superior to others in some of the environments but not all. Multiple linear regressions can be implemented to describe a trend between a genotype and environments **(D)**: a logarithmic or polynomial adjustment (grey line) or a broken-stick adjustment (black line) (adapted from Bodin et al., 2015). The broken stick model identifies the point at which increasing the environmental factor (e.g., the provision of straw as enrichment) no longer affects the behaviour (e.g., tail biting).

Damaging behaviours are influenced by both genetic and environmental factors, and therefore interactions between the genotype of a focal individual and the environment in which it is raised and/or housed (GxE) must be considered. Studies of GxE address the variation in relative performance of two or more genotypes between different environments. Thus, two genotypes showing the same amount of damaging behaviour in one environment may show markedly different amounts in a second environment (Figure 2). This may result in different genetic parameters estimated either across populations or within a population raised under different environmental conditions. Within the herd, the recording of animal movements between pens and buildings is essential information, so that the identity of the group of animals in which a focal pig is raised is known at any time and can be accounted for as an environmental factor in the analysis. Group size can be derived from these data and needs to be included as well, since it may interact with the genetic expression of damaging behaviour.

GxE has been observed across a wide range of behavioural traits, including behavioural indicators of coping with stress and cognitive ability (Shanahan and Hofer, 2005). Initial studies in finishing pigs showed no GxE for exploration, and aggressive and non-aggressive biting considered separately (Hill et al., 1998; Guy et al., 2002). A more recent study highlighted strong GxE for the receipt of tail bites (Canario and Flatres-Grall, 2018). Indications of GxE for damaging behaviours mean that conclusions from genetic studies must be drawn with care. Indeed, given that variability in behaviour is influenced by environmental factors, a genotype can be used for production in an environment for which it is not adapted, and as a consequence the effectiveness of selection can be reduced (Bowman, 1972).

## Models for the Analysis of Damaging Traits

The modelling of damaging behaviours relies on standardised recording of behaviour in a whole population, or a sample population, and the matching of these data with pedigree information to run genetic analyses.

### Analysis With a Classical Quantitative Genetic Model: The Case of Piglet Savaging

We assume that piglet savaging has a quantitative genetic background with many genes involved, all with a small, additive effect on its expression. Savaging is regarded as a trait of the sow, and thus the sow is “the animal” in the animal model. If it is recorded as the number of killed piglets, or as the percentage of piglets killed in the litter, a linear model can be used to estimate genetic parameters and breeding values for it. In the linear model, the record (0, 1, 2, … n killed piglets) is the sum of the genotype and the environment. By setting up an equation for each animal and incorporating pedigree information (i.e., a relationship matrix depicting relatedness between individuals of the genetic population) we can estimate the genetic and environmental variances and breeding values. This model is far from perfect, since the number of killed piglets does not follow a normal distribution. In most cases there is no savaging, and often when there is savaging all of the piglets are killed. Furthermore, savaging (like many other damaging behaviours) is usually recorded as a categorical trait with two records: savaging or no savaging. If the records consist of only 0 and 1, the ordinary linear model will underestimate genetic variance and thus heritability.

Threshold-linear models are often used for categorical traits with a quantitative genetic background. Savaging behaviour seems to be related to low plasma oxytocin levels (Gilbert, 2001). Let us assume, in the present instance, that the hormone level is normally distributed and that only sows with a hormone level below a certain threshold perform savaging behaviour. Although this underlying variable is not observed, the genetic variance and breeding values for savaging can then be estimated based on the observed phenotypes (savaging or no savaging) and the relationship matrix. The threshold-linear model thus describes an underlying unobservable variable. Such a model is relevant for many behaviours recorded as a binary trait (yes/no), including aggression and tail biting, and in this case it can be helpful to assume a physiological threshold below or above which a change in behaviour is triggered.

Savaging is more common in first parities than it is in later parities, but older sows sometimes display the behaviour too (e.g., Gäde et al., 2008). If more than one parity is included in the analysis, a permanent environmental effect of the sow should be added to the model. This effect describes an environmental effect which influences the sow's behaviour throughout its productive lifetime but which is not a genetic effect, and thus is not inherited.

A limited number of quantitative genetic studies of savaging or “aggressive behaviour towards piglets” can be found in the literature. In the 1980s, Knap and Merks (1987) performed a paternal half-sib analysis on 987 Dutch Landrace primiparous sows and presented a heritability estimate of 0.25. The behaviour was recorded as showing “vigorous aggressiveness” or not. The threshold-linear model was not yet developed at that time, but when it was corrected for the categorical nature of the records the heritability of the underlying trait was estimated at 0.87. van der Steen et al. (1988) studied aggressive behaviour towards piglets at parturition in two groups of around 900 primiparous sows. They also used a paternal half-sib analysis leading to a heritability estimate of 0.1–0.2. Gäde et al. (2008) analysed records from 10,657 sows (16,012 farrowings) with a threshold model for repeated records. The heritability was low (0.02) but the repeatability was high (0.41). Vangen et al. (2005) used questionnaires, answered by farmers, to estimate heritabilities of sow behaviour. One question was “How much aggressive behaviour does the sow show against her piglets at farrowing?” This question was answered on a scale from 1 (very much) to 7 (nothing). Around 55% of the sows showed no such behaviour and the heritability was estimated close to zero (Vangen et al., 2005).

Theoretically, given that both the sow and the piglets play a part in the outcome of an attack (e.g., whether the piglet is killed may depend on its ability to escape attack), the genotype of the piglet could influence the risk of being savaged. It seems likely that vigorous, heavy piglets have a better chance of escaping an attacking sow. Grandinson et al. (2003) found that the average birth weight of savaged piglets (*N* = 419) was 200 g lower than the average for all piglets (*N* = 11,016). To account for the influence of both sow and piglet genes on savaging, two genetic effects—the direct genetic effect of the piglet and the maternal genetic effect of the sow—can be included in the model. Such a direct-maternal model (often used for piglet weight, e.g., Lundgren et al., 2010) parallels the social model in which the identity of several pigs kept together in the same group is included. We were unable to find any studies in which savaging is analysed in a direct-maternal model.

### Social Network Analysis Applied to Pig Behaviour

**Figure 3:**
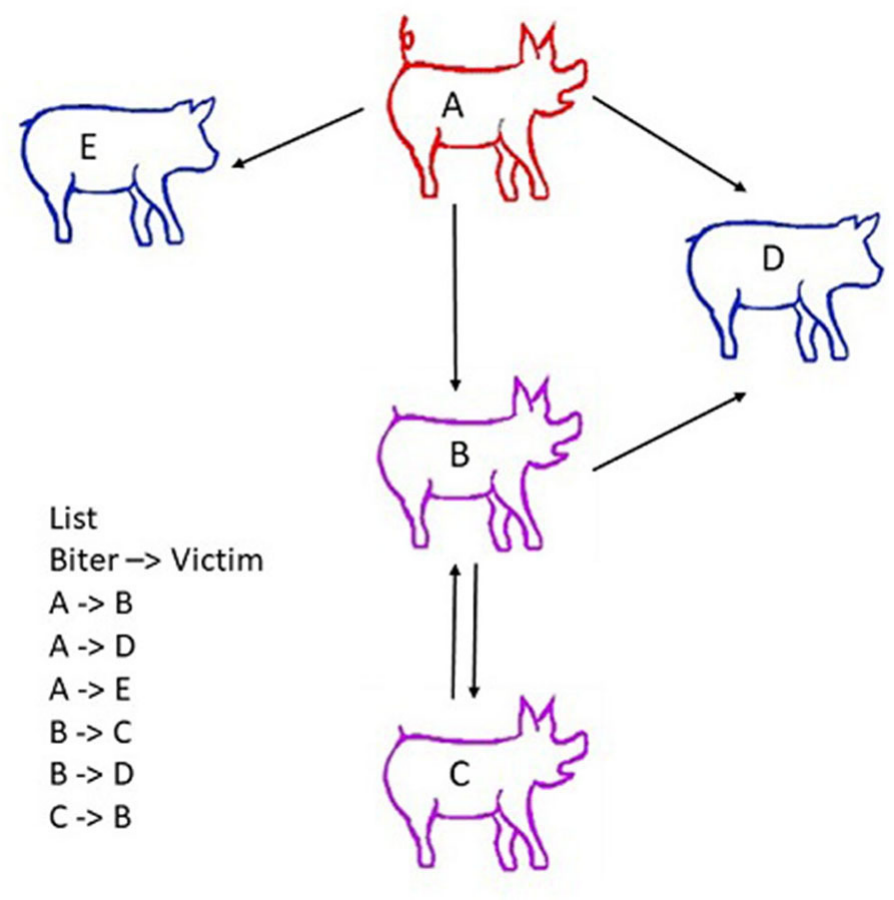
Scheme describing the principle of social network analyses (SNA). Social network analysis (SNA) accounts for both direct interactions between animals (e.g., pig A bites pig B) and indirect interactions (e.g., A bites B, B bites pig C, and therefore A and C have a relationship through B). Victims are indicated in blue, the biter in red, and a pig that is both aggressor/biter and victim in purple. Nodes represent individual animals. Edges represent interactions between them. An edge list is created from observations on farm, video analysis or the tracking of animals. The list of all dyadic encounters is established (i.e., A attacks B; C attacks B, etc., …). This edge list includes all single dyadic encounters observed. It may encompass a number of repeated dyadic encounters between two pigs, and the centrality measures derived from the dataset may be weighted to account for these repeated interactions. To facilitate statistical analysis, the edge list is summarized in a matrix of social interactions called a sociomatrix or adjacency matrix (Wasserman and Faust, 1994).

Farine and Whitehead (2015) provided a general framework for applying social network analysis (SNA) in the animal context in order to model social interactions within a group. The model incorporates both direct interactions between animals (e.g., dyadic encounters where one pig bites another pig) and indirect interactions where a pig has an intermediate role between focal pigs (i.e., pig A bites pig B, B bites pig C, and therefore A's behaviour has an impact on C via the intermediate B, as illustrated in Figure 3 (Asher et al., 2009; Büttner et al., 2015a,b). It therefore assumes that individuals are interdependent, that dyads do not interact in isolation from the rest of the social group, and that the behaviour of a member of the group could influence other members' behaviour and performance (Büttner et al., 2015a). The main advantage of SNA is to quantify the relative position of individuals (Goh, 2002; Strandburg-Peshkin et al., 2013). It provides a means of linking social behaviour across all levels of organisation, from the individual, to subgroups, to the whole social group. In that respect, it appears to be an ideal method of describing the structure of social relationships in pig groups, and of analysing damaging behaviours in pigs, including levels of aggression and tail, ear and vulva biting.

Kleinhappel et al. (2016) have recently emphasized the potential value of SNA in analysing the spread of damaging behaviours such as tail biting, because it is still unclear how these kinds of behaviour are transmitted. They suggested that comparing social networks over time might help to gain more insights into how such behaviours spread. The main objective would be to identify aggressors/biters and victims, to explain how these roles are associated with position in the social network, and to identify key-individuals. The impact that one individual has on the group, and vice versa, can be quantified by removing it from the network. The latter can then be examined with egocentric networks (Scott, 2017) that enable a better understanding of group hierarchy (Shizuka and McDonald, 2012). This information, though useful at the experimental level, would not be used for routine breeding.

To perform social network analyses, information listing all dyadic interactions that occur in the group over the study period (e.g., pig A attacks pig B, pig C attacks B, and so on) is needed. Nodes and edges depict the relative positions of individuals in the network; animals are regarded as the nodes, and edges represent interactions between two individuals. Edges frequently reflect the quality of the relationships, including affiliation between nodes and frequency of interactions (Farine and Whitehead, 2015) and/or aggression (Makagon et al., 2012; Büttner et al., 2015b; Foister et al., 2018). Graphs are usually constructed in such a way as to enable the interconnections between nodes and the entire structure of the network to be visualised. Edges may be considered as binary elements, where 0 is assigned in the absence of an interaction, and 1 is assigned when at least one interaction has occurred between two pigs (Wasserman and Faust, 1994; Wey et al., 2008). In weighted networks, differences in the line thickness of edges represent variation in the strength of the relationship (e.g., frequency or duration of interactions) between pairs of pigs, and in a directed network, arrows display the direction of the interactions between pigs (Newman, 2004; Farine and Whitehead, 2015) (Figure 3).

A variety of quantitative centrality indexes have been proposed by network analysers to determine the degree of importance of any node within a network (Wasserman and Faust, 1994; Scott, 2017). The centrality of individuals within the network is an output measurement depicting how important certain pigs are in the group relative to others—e.g., in eliciting attacks. Three of the most common ways to quantify the importance of a node (e.g., pig) are by using degree centrality (number of contacts), betweenness centrality (number of shortest paths between two pigs that go through the focal pig) and closeness centrality (average lengths of all the shortest paths from one pig to all the others) (Wasserman and Faust, 1994).

As these centrality measures are obtained for every individual, they can be used for breeding in an additive direct model (Fisher and McAdam, 2017). Potentially, this will have a greater impact on harmful behaviour than could be achieved from conventional records of social behaviour created merely at the dyadic level of performer or victim. The social parameters, built as a social association matrix, can alternatively be used to weight social genetic effects in order to estimate how much a pig will influence the phenotypes of others, or how much its own phenotype will be influenced by its neighbours (Fisher and McAdam, 2017).

Li et al. (2018) applied SNA to tail biting, and Büttner et al. (2015a,b); Büttner et al. (2019) and Foister et al. (2018) applied SNA to pig agonistic behaviour after mixing. The networks of agonistic interactions showed strongly connected components of more than one opponent. This emphasises the importance of going beyond dyadic analyses. In the studies conducted by Büttner et al. (2015a,b) there was also some evidence of a link between the position of an individual in the network from one growth stage to another, and of the possibility that previous experience can influence this position later on (Figure 1).

The information required to build links in a network can be obtained via direct observations of pig behaviour (Løvendahl et al., 2005), but in the case of low-frequency behaviour this method may rapidly become tedious and inaccurate. As an alternative, edge lists may be created using automatic video recording or sensors. Examples of behaviour traits recorded in feeders are feeding rate, feeding frequency, occupation time and time between visits, and they are all relevant for studies of social behaviour in groups of pigs (Ragab et al., 2019). Rank order at the feeder is heritable (Jonsson, 1985), and feeding behaviour may be associated with damaging behaviours (Wallenbeck and Keeling, 2013). As long as damaging behaviour is not easily recordable in groups of pigs, other existing sources of information, such as automatically recorded feeding data, should be explored to determine whether these sources predict the performance of damaging behaviour. In-depth analysis of these data—e.g., to explore meal patterns, or, at the network level, to classify pigs according to their feeding hierarchical position—may give us a tool to predict damaging behavioural phenotypes.

### Capture-Recapture Analysis to Impute Missing Biter and Victim States

Accurately recording the behavioural response of each individual within a group is complicated, especially when many individuals are likely to interact with each other, when the environment is complex, and when the behaviour being investigated is infrequent and of short duration. Multiple observations of an individual make it possible to study changes in its state in a dynamic way—e.g., the change between being uninjured to being a victim of harmful behaviour. In the simple case of on-farm *de visu* observations, it is possible to ask motivated farmers to observe animals individually once a day to identify victims according to lesions, and to maintain this daily characterisation over several weeks covering multiple stages of life on the farm. Electronic devices capable of individually identifying animals can replace de *visu* observation, but even sensor-derived behavioural recording is likely to include a non-reducible proportion of missing data as a result of, for example, loss of ear tags. Also, video analysis is unlikely to detect subtle behaviours such as tail or ear biting when stocking density is high, or when pens are large or include hidden areas. An animal observation may thus be missing or fail to fully reflect the animal's state. Therefore, these scattered data, which are of utmost importance in deciphering the behaviour, are only recorded with a certain level of uncertainty. Their processing may be complex (Yu and Kobayashi, 2003).

The multi-event/multi-state model was developed for handling scattered and uncertain data collected from wild populations monitored with low capture or viewing rates (Lebreton and Pradel, 2002; Pradel et al., 2005). In its simplest form, the multi-event multi-state model provides a statistical framework for manipulating event observation probabilities by considering unobserved (hidden) states (e.g., survival) modelled as a Markov chain (Carola et al., 2011; Gimenez et al., 2012; Langrock et al., 2012).

**Figure 4:**
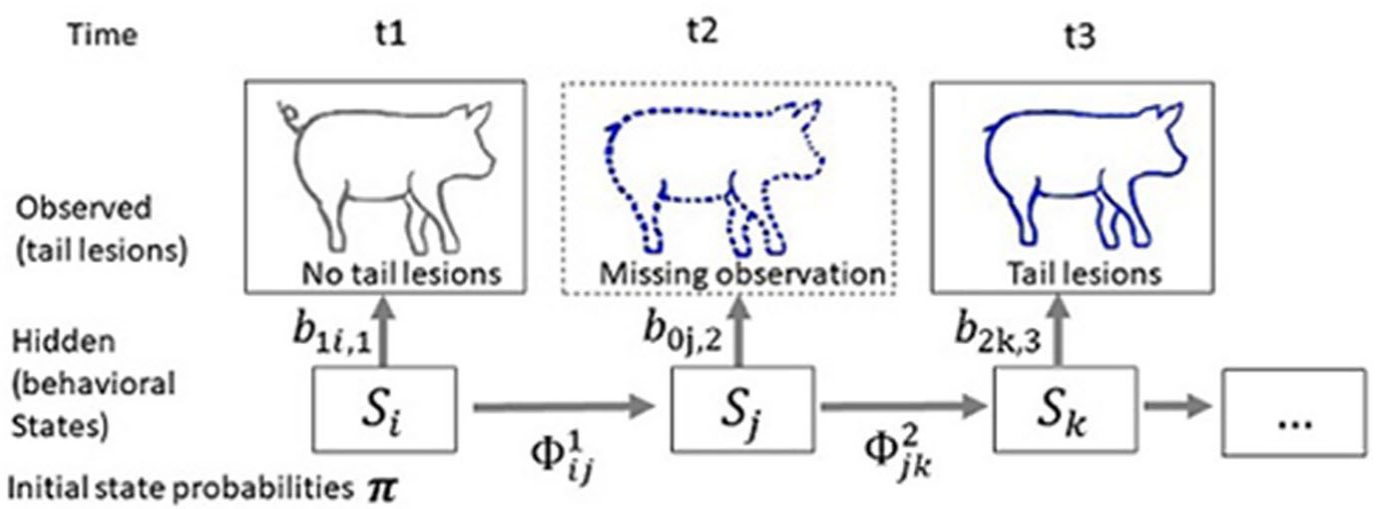
Scheme describing the principle of a multi-event multi-state model applied to the status of being a victim. The objective is to estimate pig victim status using information collected at time t1 to t3. Tail lesions are the observation (*o*_*t*_ = 1 if no tail lesion is observed at time t, *o*_*t*_ = 2 if tail lesions are observed at time t and *o*_*t*_ = 0 if the animal is not observed at time t). At first observation t1: no tail lesions are observed, while at t3 tail lesions are observed. A missing observation, indicated with a dotted line, has occurred at t2. The observed history of the animal is (102). The unobserved state (v, victim; nv, not victim) follows a Markov chain of order 1 with transition probabilities ϕ, $\phi_{ij}^{t}=\mathbb{P}\left(S_{t+1}=j|S_{t}=i\right)$ that are the probabilities that the focal pig transits between the two states of not being a victim or being a victim. The probability of observation *i* given the victim state k at time t is *b*_*ik, t*_ = ℙ(*o*_*t*_ = *i* | *S*_*t*_ = *k*). The probability of the history of the animal is then $\mathbb{P}\left(102\right)=\underset{i,j,k}{\sum }\pi_{i}b_{1i,1}\phi_{ij}^{1}b_{0j,2}\phi_{jk}^{2}b_{2k,2}$ where i, j, k span all possible states.

Tailored to the studies of damaging behaviour, the multi-event multi-state model can be illustrated as follows (Figure 4). Consider the simple case of three discrete observation times t1, t2, and t3, and suppose that we are interested in the victim states of the animal. There are two possible states (*S*_*t*_) at each time point, victim and non-victim, and these cannot be observed (i.e., are hidden). The state process is modelled by a Markov chain, generally of first order, with transition probabilities $\phi_{ij}^{t}=\mathbb{P}\left(S_{t+1}=j|S_{t}=i\right)$. For instance, if we note *v* and *nv* the “victim” and “non-victim” states, $\phi_{nv,v}^{1}=\mathbb{P}\left(S_{2}=v|S_{1}=nv\right)$ is the probability that the animal is a victim at t2 given that it was not a victim at t1. Four transitions are possible in the present illustration, $\phi^{t}=\left[\begin{array}{cc} \phi_{v,v}^{t} & 1-\phi_{v,v}^{t}\\ {1-\phi }_{nv,nv}^{t} & \phi_{nv,nv}^{t} \end{array}\right].$The unobservable states determine the distributions associated with the observations that can be performed on the animal. In the present case, tail lesions are the observation (*o*_*t*_ = 1 if no tail lesions are observed at time t, *o*_*t*_ = 2 if tail lesions are observed at time t, and *o*_*t*_ = 0 if the animal is not observed at time t). The observed history of the animal can then be reduced to the series (102). The model assumes that, given the victim state at time t, the distribution of the observations is independent of all previous states and observations: ℙ(*o*_*t*_ = 1 | *S*_*t*_ = *k*) = *b*_1*k, t*_. In the present illustration, we use the matrix $B_{t}=\left[\begin{array}{cc} b_{1v,t} & b_{1nv,t}\\ b_{2v,t} & 0\\ 1-b_{1v,t}-b_{2v,t} & 1-b_{1nv,t} \end{array}\right]$ where *b*_1*nv, t*_ is the probability of observing no tail lesions given that the animal is not a victim. The probability of observing tail lesions given that the animal is not a victim is null. The initial state probability is ****π**** = (ℙ(*S*_1_ = *v*), ℙ(*S*_1_ = *nv*)). Given these parameters, it is possible to express the probability of the history of the animal depicted in Figure 4 as $\mathbb{P}\left(102\right)=\underset{i,j,k}{\sum }\pi_{i}b_{1i,1}\phi_{ij}^{1}b_{0j,2}\phi_{jk}^{2}b_{2k,2}$ where i, j, k span all possible states. The likelihood for a population would then be the product of the probabilities of all of the histories. In the study of livestock species, mortality is not one of the unobserved states, and the initial state probability is not time-dependant since all animals are included in the study when starting a life stage or test period, and thus all are identified for the first time at the same time. This last feature introduces a substantial simplification that is not present in the initial multi-event multi-state model of Pradel et al. (2005).

To reduce the number of parameters to estimate $\left(\phi^{t},B_{t},\;\pi \right)$, and to ensure parameters' identifiability, the transition probabilities and probabilities of observation for a given state can be considered as a function of time (Schliehe-Diecks et al., 2012). It is also possible to take these parameters to be influenced by environmental factors, such as group size (McKellar et al., 2015), that would be included in the functions of the different parameters as fixed effects. Moreover, these could even be taken to be specific to each animal, using random effects as is done in Schliehe-Diecks et al. (2012). This would be of particular interest in genetic studies.

Parameter estimations can be performed by the (restricted) maximum likelihood method (Dedieu et al., 2014). However, Bayesian approaches are often preferred for complex models (Vogel et al., 2020). Outputs of the model that would be of interest in genetic studies could include the subject-specific probabilities of hidden states at each time point (these could be replaced by the most likely state for simplicity; Leos-Barajas and Michelot, 2018) and/or the subject-specific initial state and transition probabilities that depict the dynamic change of the animal's behaviour over time. New subject-specific variables extracted from this model can then be analysed using classical genetic models in a second step.

Victim states were used to illustrate, in a simple manner, the multi-event multi-state model adapted to the study of behaviour in livestock. This model exploits data from several samples taken at different points in time to estimate probabilities of states. It therefore requires temporal tracking of the animals studied, and life tracking is recommended. Determinations of the states of the animal (biter/non-biter, victim/non-victim) are also of great interest in the analysis of damaging traits. The victim and aggressive states can be viewed as independent phenomena, and thus they can be processed with two separate hidden Markov chains. However, in real social interactions, assumed independence of the two traits may be unrealistic and it would be preferable to consider states that combine the different behaviours potentially expressed by the animal. If we note b = biter and nb = non-biter, there are four hidden states: v.b/nv.b/v.nb/nv.nb. This also enables account to be taken of the fact that pigs can change status from aggressor/biter to victim over time and throughout their life stages (Ursinus et al., 2014). Also, the possibility of estimating the probability of a state with sufficient precision depends on the sampling frequency, and this must be determined according to the frequency of the studied trait. For instance, in the study of the aggressive states which are of low prevalence (say, 5%) we suggest using high frequency observations. Indeed, if the prevalence of attacks is low, but the time-lapse between observations is short, the probability of remaining in a given state between two time points of observation will be high.

A difficulty when determining the unobserved state (and one that exacerbates the difficulties of convergence of the model) emerges if the observation is not specific to a given state (i.e., when the columns in **B** are similar). In the case of being a biter, if we note the observations animal biting = 1, not biting = 2, not observed = 0, then *b*_1*na, t*_ = 0 (the probability of observing an animal biting given that it is not an aggressor/biter is null), but *b*_1*a, t*_ is also low because attacks are of very short duration. To overcome this difficulty, we suggest referring to an additional phenotype that is predictive of the biting activity. Chewing on the tail of a conspecific is often a precursor of damaging behaviour. Chewing group mates' tails, or other parts of their bodies, is more frequent than attacks (Camerlink et al., 2015). Therefore, it is advantageous to add this information in the model. The chewing activity can be considered as an additional event (3) for biter status with high value of *b*_3*a, t*_. The chewer status can also be modelled with a hidden Markov chain (corresponding to observed events: chewing group mate's tails or not) linked to the biter status under the form of a probability whereby, by definition, the probability of being a tail biter will be higher if the pig was a chewer earlier. This will facilitate use of the multi-event multi-state model and improve the completeness of the database.

### Models With Social Genetic Effects to Account for Group Mates' Identity

**Figure 5:**
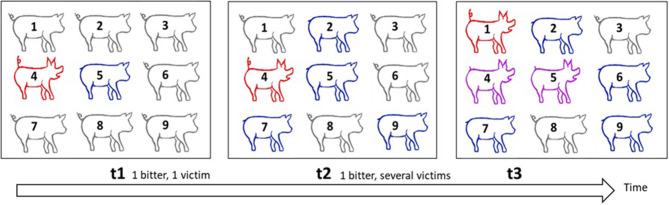
Illustration of the spread of tail-biting in a group as a form of contagion. This scheme illustrates how the contagious tail biting occurs in a group of pigs. Red signifies biters, blue victims, purple those who are biters and victims, and grey those who are neither biters nor victims. At t1, pig 4 is a biter and targets pig 5, whose tail becomes injured. At t2, pig 4 bites more victims and the tails of pigs 2, 7, and 9 are also injured. At t3, some pigs are attracted by blood and become biters themselves. Pig 1 becomes a biter, pig 6 is a new victim, pig 5 who was a victim at t2 is now also a biter. The new victim, pig 6, may have been bitten either by pig 1, 4, or 5. With several biters and victims, it is impossible to identify dyads that interact, and if the time-lag between the observations is short, tails remain unhealed and one cannot identify new bites. It therefore becomes difficult to identify at which time point the injuries occurred. Each biter is potentially a biter of newly bitten pigs. Even biters may become victims in turn.

Phenotypes can be affected through interaction in either a positive or a negative manner, and the trait value of a pig may be affected by the genotypes of other pigs. As explained above, while a direct genetic effect is the effect of an individual's genes on its own phenotype, a social (indirect) genetic effect is its effect on the phenotype of its social partners (Griffing, 1967; Moore et al., 1997). For example, assume the trait of interest is biting behaviour. The direct genetic effect is now the effect of the genes of the focal pig on its own biting activity. The social genetic effect (SGE) is the effect of the genes of the focal pig on the biting activity of its group mates. When the behaviour itself is not accessible, lesions due to biting can be observed. In that case, we combine the direct effect of showing tail lesions (reflecting being bitten) with the social effect that describes causing tail lesions on group mates (reflecting biting others). In classical genetic evaluation, a direct breeding value (DBV) describing the additive contribution of the genes of the focal pig to its own phenotype is estimated for each pig. When a social model is used, a social breeding value (SBV) is also estimated for the social effect of that focal pig on the phenotype of others (Figure 5). So far, social genetic effects have been estimated in pigs essentially for production traits such as growth (Bergsma et al., 2013; Canario et al., 2017). In these cases, the social genetic effect is most likely to be a result of unobserved behaviour. Thus, a negative social genetic effect on the number of piglets born alive of a conspecific may be explained by a high level of aggression between gestating sows (Bunter et al., 2015). Camerlink et al. (2015) and Canario et al. (2012) analysed relations between SBV for growth and DBV for a variety of behaviours including aggression in unstable and stable groups. Their results suggest that housing groups of pigs with a positive SGE on group mates' growth together reduces damaging behaviours. The advantage of this approach is that it requires no behavioural phenotyping and behaviour may be improved indirectly as a consequence of improving growth. Conversely, the specific targeting of improvements in damaging behaviour as a response trait using social genetic models will require new phenotyping tools for behaviour traits enabling access to the large databases of individual records that are required for the convergence of a social model.

When a classical direct model is applied to traits depending on social interactions, the extra heritable variation that is attributable to social genetic effects is partially hidden in the residuals, and in other variances such as the environmental group effect that accounts for the identity of the group in which the focal pig is raised. A way of disentangling group effects of non-genetic and genetic origin in a social model is explained in Muir (2005), Bergsma et al. (2008), Bijma et al. (2007), and Bijma (2014). Models integrating both direct and social sources of genetic variation have proven superior in capturing genetic variation (Ellen et al., 2014). The comparison of classical heritability (h^2^) with the total heritable variance relative to phenotypic variance (T^2^) that accounts for social variation allows the contribution of social genetic effects to heritable variation to be quantified. For instance, the heritable variance detected for pig growth can be substantially greater than it is within a classical model (Bergsma et al., 2008; Canario et al., 2017). With regard to the receipt of tail biting, as measured by tail lesions in finishing gilts, large social genetic effects contributing 81–93% of total heritable variance were detected. These effects equated to between 40 and 80% of phenotypic variation, whereas direct effects explained only 6% of that variation (Canario and Flatres-Grall, 2018). Therefore, social structure and the resulting interactions among group mates play a major part in the genetic expression of this proxy for tail biting receipt.

The model accounting for social genetic effects includes genetic covariance between direct and social effects, leading to a direct-social genetic correlation. The interpretation of the latter is important in understanding population functioning, and in selection decisions (Bijma et al., 2007; Rydhmer and Canario, 2014), see Box 1. The correlation is negative (unfavourable) if pigs compete for limited resources such as food and space. If this is the case, selection based only on high DBV will be detrimental to the group members, reflecting the negative correlation between direct and social effects (*r*_*A*_*DS*__ < 0). If the genetic correlation is positive (*r*_*A*_*DS*__ > 0), cooperation occurs and selection of the animal with the highest DBV will not harm group members. In this case, accounting for social genetic effects would also yield an advantage in the selection response, as compared with that obtained in a model with only the direct effect (Ellen et al., 2014). The role of this correlation in damaging behaviour can be illustrated with simulations of different situations in which the pigs display a negative covariance between direct and social effects for growth (genetic antagonism between own growth and the growth of group mates; (*r*_*A*_*DS*__ < 0) through to a positive covariance (genetic mutualism between own growth and the growth of group mates; *r*_*A*_*DS*__ > 0) (Canario et al., 2012). Under conventional pig rearing conditions, the direct-social genetic correlation is often close to zero (see Rydhmer and Canario, 2014 for a review of the extended range). In that case, pigs with high SBV for growth lose more fights and receive more aggression at mixing, and pigs that are selected to have both high DBV and high SBV for growth initiate more bullying after mixing and are less willing to fight 3 weeks later in a more hierarchically stable group. In addition, null direct-social genetic correlations for tail biting receipt were estimated in two populations of the same line but with a different prevalence of tail bites (6.6 and 10.8%), and a different average group size (12.7 and 14.7 pigs) and feeding system (Canario and Flatres-Grall, 2018). The neutral associations meant that gilts with a high genetic merit for being a victim of tail biting (high DBV) did not have a high SBV. Thus, the probability of other gilts in their group being tail bitten would not be increased by selecting against a high DBV.

Box 1 **Genetic (co-)variances in the social model.**

A stepwise procedure is often used to test the significance of social genetic effects. Data can be analysed using the restricted maximum likelihood methodology, as implemented, for example, in the ASReml software package (Gilmour et al., 2015). First, an initial model with direct genetic effects only is fitted to the data: **a**_*D*_ is a vector of direct additive genetic effects of the focal individual, with normal distribution of mean 0 and variance **A** $\sigma_{A_{D}}^{2}$where **A** denotes the matrix of additive genetic relationships between pigs and $\sigma_{A_{D}}^{2}$the direct additive genetic variance. In addition, a vector of random group effects with normal distribution of mean 0 and variance connected to an identity matrix is considered. With social interactions, the model is extended with random social (indirect) genetic effects of group mates, following the methods outlined by Muir (2005), Bijma et al. (2007), and Bergsma et al. (2008). This extended model includes a known incidence matrix linking group mates to the record of an individual through the value of 1 for each group mate of the focal pig, with **a**_*S*_ as a vector of social genetic effects. The social additive variance $\sigma_{A_{S}}^{2}$refers to the variance of a social genetic effect expressed on a single recipient. The model also accounts for covariance between direct and social genetic effects, using a variance structure with multivariate normal distribution of means 0 and a matrix connecting the direct variance, the social variance and an estimated direct-social covariance with **A**, in the form of $\left[\begin{array}{c} {\text{a}}_{D}\\ {\text{a}}_{S} \end{array}\right]\tilde{\;}MVN\left(\begin{array}{c} 0\\ 0 \end{array},\left[\begin{array}{cc} \sigma_{A_{D}}^{2} & \sigma_{A_{DS}}^{\;}\\ \sigma_{A_{DS}}^{\;} & \sigma_{A_{S}}^{2} \end{array}\right]⊗\text{A}\right)$. The sign of the covariance between direct and social genetic effects (σ_*A*_*DS*__) determines the sign of the correlation between the direct and the social genetic effects, i.e., the direct-social genetic correlations (*r*_*A*_*DS*__) (Bijma et al., 2007).

### Longitudinal Models With Social Genetic Effects to Analyse Contagion

At present, no quantitative genetic models to study social interactions and injuries over time are available. We shall present a sketch of how such models could be developed, borrowing from models for the transmission of infectious diseases and from survival time analysis (e.g., Ducrocq, 1994; Anche et al., 2014; Lipschutz-Powell et al., 2014; Biemans et al., 2017), using tail biting as an example. In survival time analysis, the survival of individuals is modelled over time, and depends on the so-called hazard of the individual. Hazard reflects the probability that an individual will die, given that it is still alive (Ducrocq, 1994). The way tail biting progresses in a group has similarities with the spread of infectious diseases (Bracke et al., 2018) (Figure 5). From a single biter, the number of pigs that perform the behaviour increases rapidly as a result of social learning and attraction to injured tails. The probability that a focal pig will have both the status of biter and victim rises as the number of victims increases. As we noted earlier, an individual's propensity to receive tail biting is analogous to disease susceptibility, while its propensity to express tail biting is analogous to infectivity (the tendency to infect others). Moreover, as in survival analysis, and as described in the previous section, the degree of tail biting behaviour may vary over time. Hence, tail biting may be modelled as a continuous function of time, like the hazard function in survival time analysis (as used in Chou et al., 2019).

Like survival and the transmission of infectious disease, tail-biting may be modelled over time as a Poisson process. The Poisson process is commonly used to model events that may happen randomly at any point in time. When modelled as a Poisson process, the biting an individual expresses or receives is given by a (stochastic) rate, say λ, also known as a probability per unit of time. This rate is analogous to the hazard in survival time analysis. For example, with a rate of λ = 0.5 bites/day, a pig receives on average 1 bite per 2 days. The average time until the next bite equals 1/λ, which is 2 days in this example. The number of bites an individual receives in a time-slot of Δt days follows a Poisson distribution with mean λΔt. For example, with λ = 0.5 bites/day, a pig receives on average 3.5 bites per week (Δ*t* = 7 days). However, because biting is modelled as a stochastic process, there is also a probability that an individual will not receive any bites in a week. This probability follows from the Poisson distribution and equals *P*(*k* = 0) = *e*^−λ*Δt*^. With λ = 0.5 bites/day, the probability equals 0.03 (3%) in the current example. Following Anche et al. (2014), we can include genetic variation among individuals in their propensity to receive and perform tail biting. This can be done by specifying a biting rate for every pair of individuals. For focal individual *i* exposed to its pen mate *j*, the pair-wise biting rate may be defined as λ_*ij*_ = λ_0_*D*_*i*_*S*_*j*_, where λ_*ij*_ is the rate of biting that focal individual *i* (victim) receives from its pen mate *j* (biter), λ_0_ is the population average pair-wise biting rate, *D*_*i*_ is the direct effect of the focal individual *i* (victim effect), *S*_*j*_ is the social effect of its group mate *j* (biter effect), and the average values of *D* and *S* are ~1 ($\bar{D}\approx \bar{S}\approx 1$). For example, an individual with *D* = 2 receives twice as many bites as the average number, while an individual with *S* = 0.8 gives 20% fewer bites than average. With an average pair-wise biting rate of λ_0_ = 0.1 bites/day, an individual with *D*_*i*_ = 2, exposed to a group mate with *S*_*j*_ = 0.8 receives on average 0.1 × 2 × 0.8 = 0.16 bites per day from its group mate *j*. Since each individual may both give and receive bites, each individual has both a D-value and an S-value, in a similar way to the classical IGE model.

Because the number of bites cannot take negative values, *S* and *D* must be positive values. This can be guaranteed by using a so-called log-normal distribution rather than the usual normal distribution. To obtain a log-normal distribution, we use $D_{i}=e^{A_{D,i}}$, and $S_{i}=e^{A_{S,i}}$,

where *A*_*D*_ and *A*_*S*_ are normally distributed DBVs and SBVs, with a mean of zero. Thus, *D* and *S* are log-normal, positive values, and have a mean value of ~1 (since e^0^ = 1). The breeding values can be interpreted, approximately, as percentages. Thus, an individual with a DBV of *A*_*D,i*_ = +0.1 has *D*_*i*_ ≈ 1.10 and therefore receives ~10% more bites than the average individual. A log-normal distribution is skewed to the right, indicating that some individuals may perform or receive excessive biting, in line with empirical observations (Broom and Fraser, 2007; Taylor et al., 2010).

Next, the total number of bites an individual receives is the sum of the bites received from each group mate,

$$\begin{array}{l} \lambda_{i}=\overset{n-1}{\underset{j=1}{\sum }}\lambda_{ij} \end{array}$$

where the summation is over the *n*-1 group mates *j* of focal individual *i*, with *n* denoting group size. Substituting the above expressions for direct and social effects yields an expression for the rate of biting SBVs of its group mates,

$$\begin{array}{l} \lambda_{i}=\lambda_{0}\;e^{A_{D,i}}\overset{n-1}{\underset{j=1}{\sum }}e^{A_{S},j} \end{array}$$

Suppose we have groups of 4 pigs and a mean pair-wise biting rate of λ_0_ = 0.1 bites/day. Then an average pig in an average group receives (4–1) × 0.1 = 0.3 bites per day. If the focal individual has *A*_*D,i*_ = +0.1, and its three group mates have *A*_*S*_ = +0.2, −0.15, and +0.3 then the focal individual receives on average $\lambda_{i}=0.1\;e^{0.1}\left(e^{0.2}+e^{-0.15}+e^{0.3}\right)≅$ 0.38 bites per day. Moreover, the number of bites per day the focal individual receives follows a Poisson distribution with mean 0.38.

Because this model is non-linear and the number of bites an individual receives does not follow a normal distribution, an ordinary linear mixed model for analysing bite number is not statistically suitable. Box 2 summarises a generalised linear model (GLM) that can be used to analyse bite number.

Box 2 **Longitudinal analysis of bite number, taking account of social genetic effects.**

Assuming that bite number follows a Poisson distribution, we can fit a generalised linear mixed model with a log-link function. Application of the log-link function yields the following linear model (see Biemans et al., 2017 for details),

$$\begin{array}{l} log\left(E\left[y_{i}\right]\right)\approx \ln\left(\lambda_{0}\right)+A_{D,\;i}+\;\frac{1}{n-1}\overset{n-1}{\underset{j=1}{\sum }}A_{S,\;j}+\left[\ln\left(n-1\right)+\ln\left(\Delta t\right)\right] \end{array}$$

where E[*y*_*i*_] is the mean number of bites the individual receives during a time interval of Δ*t* days, ln(λ_0_) is an intercept fitted as a fixed effect, *A*_*D,i*_ is the direct breeding value of the focal pig, $\frac{1}{n-1}\overset{n-1}{\underset{j=1}{\sum }}A_{S,\;j}$ are the social breeding values of each of its group mates, multiplied by a cofactor 1/(*n*−1), *n* is the group size, and the term *ln*(*n*−1) + *ln*(Δ*t*) is a so-called offset, a known value that is subtracted from the dependent variable. As usual, the direct and social breeding values are random effects following a bivariate normal distribution with zero mean. The offset is needed only when group size varies and/or when the time interval Δ*t* varies among records. The term ln(*n*−1) in the offset assumes that the number of bites a pig receives is proportional to its number of group mates (*n*−1). If this assumption is too strong, one may drop ln(*n*−1) from the offset, and fit a fixed group-size effect instead.

This model can be fitted in standard software for generalised linear mixed models, such as ASReml (see Biemans et al., 2019 for an example; Gilmour et al., 2015). The interpretation of estimates is as follows: *e*^*intercept*^ is an estimate of the base line hazard λ_0_, i.e., the average number of bites an average pig receives per day (assuming Δ*t* is expressed in days). The relative breeding value for the rate at which a pig receives bites follows from $D_{i}=e^{A_{D,\;i}}$, where a value >1 means that a pig “attracts” more biting than the base line rate λ_0_ (which is to say “more than average”). The relative breeding value for the rate at which a pig inflicts bites on others follows from $S_{i}=e^{A_{S,\;i}}$, with similar interpretation. Anche et al. (2014) (Equations 7 and 11) explain how direct and social breeding values can be combined into a total breeding value.

If we only have data on whether (1) or not (0) a pig has received bites over a certain time interval—e.g., based on changes in tail status in an interval—we can fit the same model but then use a complementary log-log link function (Biemans et al., 2017). Hence, we use *cloglog*(*E*[*y*]) rather than log(*E*[*y*]), and *E*[*y*] is now the mean of the 0/1 trait. The interpretation of the estimates is the same as above. It is, however, important to remember that records of the number of bites are much more informative than 0/1 records.

To account for variation in tail biting over time and between environments, the intercept may be replaced by a “herd-year-season-effect”—e.g., by a separate fixed effect for each interval in each environment. When biting is observed over a longer time interval, the intercept may be fitted as a function of time using, for example, the Weibull function. Furthermore, because the same pigs are observed repeatedly, a permanent random animal effect should be included, and a random group effect is required to ensure that the social genetic effect is not overestimated (Bergsma et al., 2008). When tail-biting tends to increase over time in specific pens, as if it were a contagious social behaviour, a group*time random covariate (i.e., a random regression of the group effect on time) can be fitted to account for non-genetic covariances among group mates.

### Methods for the Detection of Genotype by Environment Interactions

In general, the consideration of environmental factors in genetic analyses is not very precise. Herd characteristics possibly involved in the causation of a damaging behaviour can be accounted for in a herd-season effect. This factor summarises information on feeding system, ambient conditions (temperature, ventilation, etc.) and enrichment (straw provision, manipulatable objects, etc.) applied at herd level. Tracking such detailed information at the different stages of a pig's life helps in the analysis and interpretation of results.

When the environment can be described as a gradient—e.g., from low to high, poor to rich, or unfavourable to favourable—the expression of a behaviour along the gradient can be presented as a reaction norm describing potential expressions of its genotype across varying environments (Johannsen, 1911). Reaction norms can be used, either on raw data or after modelling the influence of the environment on a damaging behaviour, with the aim of highlighting any environmental factors, such as herd identity, that influence the genetic expression. When the environment cannot be described as a gradient, the expression may be described as a series of character states, i.e., values as points on a curve (de Jong, 1995). Although reaction norms are mostly described as linear relationships, they can take any shape. In addition, response patterns may be dependent on threshold values that trigger or significantly modify the response pattern, resulting in, for example, broken-stick patterns, as described by Bodin et al. (2015), who examined the time spent by pigs in manipulative behaviour towards straw as a function of the amount of straw provided (Figure 2).

In the case of damaging behaviour, environmental gradients may encompass factors directly influencing the social context of the group in which the focal individual is raised (e.g., group size, feeding, stocking density). To account for GxE in the analyses, phenotypic data from a genetic line or breed needs to be collected in at least two different environments or under different environmental conditions or treatments. GxE has been considered in a one-generation selection experiment in which the biting behaviours of groups of pigs selected for low or high SBV for growth were compared in contrasting environments (housed in either conventional barren pens or pens enriched with straw and wood shavings (Camerlink et al., 2015). No major GxE effects were found. Tail damage and biting, which were much lower in the enriched pens, were almost equally affected by the housing conditions in two genetic groups formed with pigs of low and high SBV for growth, respectively. Groups of more social pigs (i.e., with higher SBV for growth) in enriched pens showed the least biting behaviour. Less social pigs (with lower SBV for growth) in barren pens exhibited the most biting. The groups of less social pigs in enriched pens also displayed less harmful behaviour than groups of more social pigs in barren pens. This resulted in an additive, rather than interactive, effect of genetics and environment, which suggests a certain robustness of the selection method for growth across housing conditions. This emphasises that both genetic selection and enriching the environment would have cumulative effects on pig welfare.

A recent study analysed the possible impact of breeding against damaging behaviour using tail biting lesions as proxy (Canario and Flatres-Grall, 2018). The presence of tail biting lesions was analysed separately in two herds which had the same genetic line but varied in their prevalence of the behaviour, average group size, feed access, etc. Accounting for both direct genetic effects and social genetic effects, it was found that the two herds did not vary in the proportion of total heritable variation detected. However, the null ranking correlation of boars frequently used in the two environments according to their direct, social and total breeding values indicated a strong GxE effect for the receipt of tail biting. The impact of GxE on response to selection is sometimes reduced by the recruitment of sires that are less sensitive to environmental conditions. Offspring from sires with stable and advantageous DBV and SBV across environments should be chosen when implementing selection against gilts that have a genetic predisposition to allow other group mates to bite their tails. To account for GxE in the genetic evaluation, heterogeneous variances could be fitted into the model applied at the population level.

## Discussion and Prospects for the Analysis of Damaging Behaviours

Behavioural characteristics and their genetic expression can be investigated from several angles. We have presented five different models: social network analysis and the capture-recapture model to analyse the behaviour measured on farm; the direct genetic effect model and the social genetic effect model *per se*, and then extended to a longitudinal contagion model, to quantify the influence of genetics on behaviour. In addition, GxE effects need to be considered in the genetic models. The aim of this paper has been to shed light on new ways to study and estimate (genetic) variation related to damaging behaviours in pigs. In this discussion section we explore how the different models could potentially be combined in order to improve our ability to detect and accurately quantify both phenotypic and genetic variation in these hard to measure traits.

### Perspectives on Data Collection and Use for Modelling

Each of the models would benefit from more frequently collected and more accurate phenotypic data, and some of the modelling strategies (e.g., social network analysis) are entirely dependent upon the availability of data on social interactions. Recording behaviour or relevant proxy measures (e.g., tail damage) is time-consuming, and observation by humans has its limitations partly as a result of the low frequency and sporadic nature of these behaviours. The development of automated technology for recording behaviour is progressing rapidly (Wurtz et al., 2019). Various projects are examining the potential for automated detection of aggression and tail biting in pigs using the concomitant tracking of animals and identification of harmful social interactions (Oczak et al., 2013; D'Eath et al., 2018; Prunier et al., 2019). It remains a real challenge to associate behavioural records with individual identities accurately in an automized way. Reliable techniques of long-term individual identification will be essential for genetic selection implemented at the individual level. Innovative use of existing data sources with precise records of pig identity also needs to be explored to predict harmful behavioural predispositions. If, in the future, technical developments allow the detection of damaging behaviours at individual animal level, we will be able, potentially, to obtain large quantities of behavioural data, define informative phenotypes (e.g., the position of centrality of a pig in a network rather than simply the sum of its dyadic interactions) and account for both direct and social effects. The combination of methods, such as radio-frequency identification (RFID) at the feeder and video-based tracking of identity, may improve the feasibility and accuracy of individual identification.

For estimating social genetic effects, and equally for the contagion model and social network analysis, it is essential to have information on which animals were housed together in a pen. A random group effect representing group identity must be included in the model if social effects are to be estimated properly. If an animal (e.g., an injured pig) is removed from the group, this needs to be recorded along with the date. In practice this takes little time, but removal is often not registered. The use of electronic tags to follow animals as they are moved across different pens or buildings on the farm has been tested in experimental herds. Its expansion to commercial herds will facilitate the recording of animal movements. Since each pen has its own specific micro-environment which may contribute to damaging behaviour (ventilation, etc.), recording of the identity of the pen will help to disentangle the effects caused by the group mates and those caused by the physical environment. Intrinsic phenotypes such as states related to growth, and extrinsic factors such as feeding system, must be recorded if behaviours are to be contextualised and analysed properly. How previous life stages may influence damaging behaviours is becoming a key question. Longitudinal records over the lifespan are needed to account for previous rearing conditions and past social experiences, with a particular interest in early-life effects (Prunier et al., 2019). Repeated recording of group identities gives access to group composition according to previous mixing, a key determinant of social interactions in the group.

### Integrating the Models

**Figure 6:**
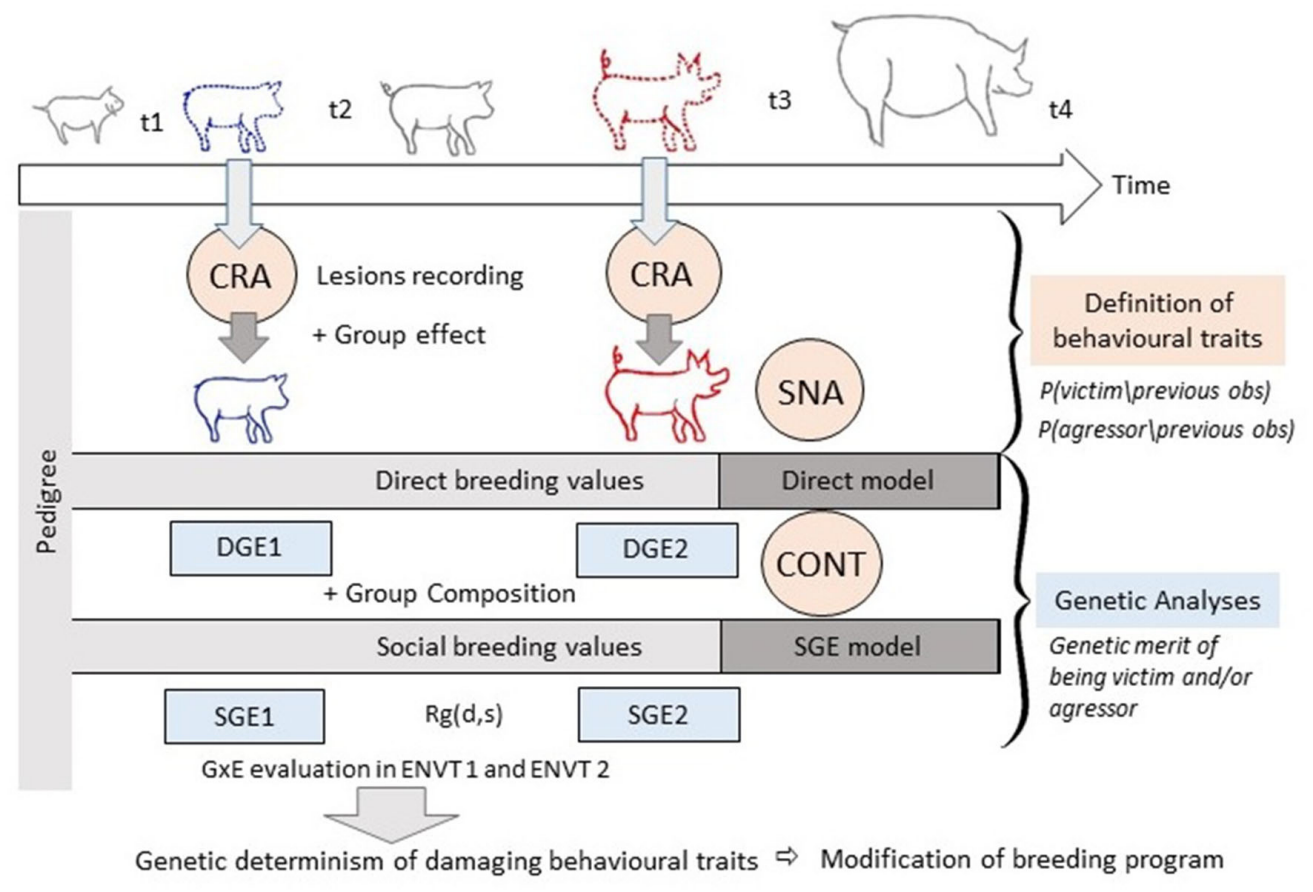
Integration of models in the frame of a lifetime genetic study of damaging behaviour. Pigs are observed at several points in their life for behaviour and lesions, and group identity is recorded at each time point: t1 is the first record after birth, t2 is a record in the growing period, t4 at the beginning of the first lactation. Missing observations are shown by pigs in dotted lines (e.g., at weaning and at the beginning of the finishing period t3). At t4, the primiparous sow may be aggressive towards piglets. The scheme illustrates the way in which models can be used successively or combined for a lifetime genetic study. Pedigree observation is available for each pig from birth, providing information on its ancestors' identity. At different time points in life, information on the group composition (identity of conspecifics) enables social genetic effect models to be used. In the upper part of the figure the behavioural traits to be analysed are defined. Methods for their analysis are indicated in the circles as follows: CRA, capture-recapture analysis to replace missing observations by imputed biter or victim states. This analysis allows missed information to be retrieved; SNA, social network analysis to estimate centrality parameters; CONT, contagion analysis to analyse the spread of injury. CONT can be used on records or combined with use of a social genetic effect model. CRA and SNA data can be used as input in the genetic model. The SNA matrix can also be treated as a social matrix and used to adjust the analysis of a trait at time *t* for the social network established concomitantly or on previous occasions. At each time point, direct and social breeding values of each pig can be estimated. With the association of observations at different time points, the probability of a pig being a biter, if its previous states are known, can be calculated. Similarly, the genetic merit of a pig being a biter at time *t* can be estimated using information available from other previous occasions.

With the current rapid developments in computer vision, sensor technology and automated detection with deep learning algorithms, large-scale automated collection of longitudinal data on damaging behaviours and injuries may become available within a few years. Combining longitudinal and genomic data should facilitate accurate estimation of breeding values for social behaviour. Under the assumption that data on behavioural phenotypes will become available, we shall now make several suggestions as to how the models could be combined and highlight a number of associated benefits (Illustration Figure 6). Pedigree information is available from the breeding companies and direct genetic effects are routinely estimated for performance traits, often several times during the focal animal's lifetime. Direct breeding values can also be calculated from behavioural observations directly, as has been done for aggression (Løvendahl et al., 2005; Turner et al., 2006) and tail biting (Breuer et al., 2005). In the cases of SNA and CRA, models can be complemented with a pedigree relationship matrix to address the heritable variation of damaging behaviours. More simply, outputs from SNA and CRA models can be used as inputs for classical animal models (for SNA: Foister et al., 2018). Datasets on damaging behaviour are often punctuated by missing data and observations that are an imperfect translation of the states of the animals. It is in filling these gaps that multi-event multi-state models can play a role by allowing unobserved states to be imputed, thereby providing more data that can be used in the other models. The CR model can therefore be used as a step between data collection and the application of the social genetic effect model, SNA or contagion model.

Selection on social breeding value, describing the overall positive effect on others, might be a more effective way to improve animal welfare than direct selection against specific behaviours. Some research reveals both an SGE originating from the biter and a direct genetic effect originating from the victim, and both effects contribute significantly to the total heritable variation. This indicates that a predisposition to receive biting may easily be overlooked in direct behavioural observations, whereas selection for the combination of direct and social breeding values utilises the full heritable variation underlying the trait. In their review, Fisher and McAdam (2017) underlined the similarity of the goals for studies with SNA and SGE, i.e., to determine the interactions between one individual and its social partners and their influence on the individual's phenotype. The authors describe a simple model to implement social interactions as a random effect in the classical animal model, accounting in this way for a social interaction matrix. In that respect, techniques developed in SNA would be highly relevant. The next step would be to integrate SNA with SGE in a single model.

The classical SGE model assumes that an animal's social effect is similar on all other group mates, regardless of whether they are relatives or not, or familiar or not, and irrespective of how much each dyad interacts. If social models currently assume that one animal has the same effect on all other animals, and that this effect relies only on the identity of group mates, access to individual behaviours and the combination with SNA will overcome this limitation. If animals do not interact equally, the model could be refined in line with developments described by Alemu et al. (2016) and Ragab et al. (2019). Within a group, animals tend to behave differently towards strangers than towards familiar individuals. Alemu et al. (2016) considered that the presence of kin-specific behaviour may complicate the selection for SGE, because social genetic effects on kin may differ from those on unfamiliar individuals. They extended the classical SGE model to allow kin and non-kin interactions to differ. Ragab et al. (2019) showed that including feeding behaviour, especially daily time in the feeder, improved the fit of the SGE model. Using records from the feeders in the social model to define the degree of interaction between group mates will thus result in more accurate direct and social breeding values for growth rate. They incorporated the pairwise intensity of social interactions in the SGE model based on Euclidean distance between animals computed from feeding behaviour traits. Theoretically, it is also possible to quantify the interactions in a social interaction matrix and add that matrix to the social model (Steibel et al., 2017). This would be meaningful in accounting for social interactions in the analysis of performance traits such as growth rate. The social interaction matrix has not been tested on real animal data yet, but it may lead to more accurate estimation of social genetic effects. Genomic selection may be particularly relevant for behavioural traits because they are regulated by many genes, each having a small but additive effect on the phenotype (Ellen et al., 2014). It can improve the accuracy of direct and social breeding values, as shown on growth rate (Hong et al., 2017; Poulsen et al., 2020). It is still little studied because genomic selection for social genetic effects requires genotyping of almost all pigs in the pen (Duijvesteijn, 2014).

Tail biting is typically seen in outbreaks (Chou et al., 2019) and can be compared with a disease outbreak which suddenly appears, spreads within a population, and then disappears. Tail biting has been described as contagious in that the number of biters increases as a result of attraction to the blood of a victim (Broom and Fraser, 2007). The contagion model could be applied to centrality measures estimated by SNA at different time points in order to model the way each animal contributes to the dynamic spread of biting within a group. Schneider et al. (2017) assessed the importance of taking into account the dynamics of social interaction in examining SGE. Potentially, this could be implemented with any type of model, with use of longitudinal analyses on a relatively short timescale (i.e., at a given stage), and thereafter across all life stages, to account for temporal effects. Undoubtedly, the longitudinal characterisation of tail biting will help to improve our understanding of the factors leading to this damaging behaviour. In quantitative analyses, a large number of records are needed to obtain estimates with high accuracy. To be efficient, the fusion of models *per se* might need even larger databases.

First, the study of damaging behaviour at a specific life stage in a pig's life, with multiple records to improve the classification of pigs in different categories, such as biter vs. victim, is recommended. This will account for the history of the behaviour in the group and thus fill in missing records (CRA model). Second, since the likelihood that an individual expresses or receives biting may depend also on its experience at earlier stages of its life, longitudinal studies may provide more insight than cross sectional studies. It is important to keep track of all animal movements between groups when integrating life information in longitudinal genetic studies. If the combination of models is preferable, offering significant added value, computational difficulties will be exacerbated by longitudinal analyses. However, difficulties associated with the low-frequency of these behaviours might be alleviated as records across a pig's life are accumulated.

A possible limitation to the implementation of all of these models at large scale arises from the need to account for GxE effects when they have a substantial impact on behavioural traits. It will be necessary to characterise the way in which different genotypes perform under different environmental conditions in more detail. Methods to investigate GxE are available, from simple comparison of re-ranking to estimation of genetic correlations among environments (Rauw et al., 2017). To date, no studies have been specifically designed to systematically allocate the offspring of each sire to contrasting environmental conditions in order to quantify GxE on damaging behaviours. This exercise will need to be performed before breeding can be adopted. Methods to account for GxE effects in breeding programmes ought to be developed. It is important to know whether the effects of genetic selection—e.g., including or not including SGE for a certain behavioural trait—are consistent and would apply to a large range of environments that differ in available resources such as feed and space.

Group size can have an impact on the prevalence of damaging behaviours, and the detection of its effect on the genetic expression of traits is a form of GxE. Until now, we have assumed that social genetic effects are independent of group size. This independence exists when, irrespective of group size, each animal is affected by the same level of social genetic effect. An example mentioned by (Bijma, 2010) is alarm-calling behaviour: here each individual receives the alarm call irrespective of how many individuals are in the group. However, many behaviours that depend on social relationships in the group are such that the social genetic effect decreases with rising group size, as each individual interacts with a smaller proportion of its group mates (Bijma, 2010). With group sizes ranging from 5 to 15 animals per pen, it has been found that the social genetic effects for growth are diluted in larger groups (Canario et al., 2017). The analysis of several populations from the same genetic line housed with different levels of food availability, stocking density, and group size, and using one or a combination of the models described above, will help to define selection strategies against damaging traits under different situations.

## Conclusion

Damaging behaviours can cause severe injuries and lead to significant impairment of animal welfare. Their prevalence can be reduced with improved environmental conditions, but it is increasingly being recognised that housing conditions as such do not eliminate all welfare problems, and that changes to housing are often limited by affordability. Genetic selection could be used as a lever to improve animal welfare by reducing the prevalence of damaging behaviours, but geneticists need better tools and methods both to harvest more data and to analyse it. With developments in automated animal identification, tracking and behavioural recording, we hope that new and improved records will be available in the near future. We have illustrated ways in which future studies can be designed, and we explained how different statistical and genetic models could be used to improve the analysis of social behaviour in group-housed pigs, with relevance to social behaviours in other livestock species. As is expected with multifactorial and complex traits, GxE effects may occur, and these should be accounted for in genetic evaluations designed to reduce damaging behaviours.

## Author Contributions

LC, PB, ID, IC, AM, WR, LZ, LF-G, ST, CL, and LR wrote the manuscript. All authors contributed to the article and approved the submitted version.

## Conflict of Interest

LF-G was employed by the company AXIOM. LZ was employed by the company Topigs Norsvin. The remaining authors declare that the research was conducted in the absence of any commercial or financial relationships that could be construed as a potential conflict of interest.
